# Trends and social determinants of the obesity epidemic among reproductive-age women in ten Asian countries

**DOI:** 10.1038/s41598-024-73522-5

**Published:** 2024-09-29

**Authors:** Subash Thapa, Kedir Y. Ahmed, Habtamu Mellie Bizuayehu, M. Mamun Huda, Binaya Chalise, Meless G. Bore, Sewunet Admasu Belachew, Tahir A. Hassen, Erkihun Amsalu, Desalegn Markos Shifti, Abdulbasit Seid, Yonatan M. Mesfin, Teketo Kassaw Tegegne, Abel F. Dadi, Daniel Bogale Odo, Getiye Dejenu Kibret, Daniel Bekele Ketema, Zemenu Yohannes Kassa, Anayochukwu E. Anyasodor, Shakeel Mahmood, Feleke Hailemichael Astawesegn, Allen G. Ross

**Affiliations:** 1 Rural Health Research Institute, Charles Sturt University, Orange, NSW 2800 Australia; 2 Health and Development Society Nepal, Lalitpur, Nepal; 3 The First Nations Cancer and Wellbeing Research (FNCWR) Program, School of Public Health, The University of Queensland, Queensland, Australia; 4 School of Nursing and Midwifery, University of Technology Sydney, Sydney, Australia; 5 School of Nursing, College of Medicine and Health Science, Hawassa University, Hawassa, Ethiopia; 6 Center for Women’s Health Research, College of Health, Medicine and Wellbeing, The University of Newcastle, Newcastle, NSW Australia; 7 Sydney Medical School, Faculty of Medicine and Health, University of Sydney, Sydney, Australia; 8 St. Paul Hospital Millennium Medical College, Addis Ababa, Ethiopia; 9 Child Health Research Centre, The University of Queensland, Queensland, Australia; 10 School of Public Health and Preventive Medicine, Monash University, Clayton, Australia; 11 Infection, Immunity, & Global Health, Asia-Pacific Health, Murdoch Children’s Research Institute (MCRI), Parkville, VIC Australia; 12 Institute for Physical Activity and Nutrition, Deakin University, Geelong, VIC Australia; 13 Menzies School of Health Research, Charles Darwin University, Casuarina, Australia; 14 Addis Continental Institute of Public Health, Addis Ababa, Ethiopia; 15 National Centre for Aboriginal and Torres Strait Islander Wellbeing Research, National Centre for Aboriginal and Torres Strait Islander Wellbeing Research, National Centre for Epidemiology and Population Health, The Australian National University, Canberra, Australia; 16 School of Public Health, Faculty of Health, University of Technology Sydney, Ultimo, NSW Australia; 17 Faculty of Medicine, Health and Human Sciences, Macquarie University, Sydney, Australia; 18 The George Institute for Global Health, University of New South Wales (UNSW), Sydney, Australia; 19 School of Public Health, College of Medicine and Health Science, Debre Markos University, Debre Markos, Ethiopia; 20 College of Medicine and Health Sciences, Hawassa University, Hawassa, Ethiopia

**Keywords:** Obesity prevention, Population attributable fraction, Reproductive-age women, Secondary education, Social determinants of health, Urbanization, Risk factors, Obesity

## Abstract

The rapid epidemiological transition in Asian countries, resulting from the rising trend of urbanisation and lifestyle changes, is associated with an increasing risk of obesity in women of reproductive age. This is the first study to investigate the trends and population-attributable fraction (PAF) of obesity, and the interaction effects of education and wealth on obesity among reproductive-age women aged 15–49 years in ten Asian countries. This cross-sectional study examined the most recent (2000 to 2022) Demographic and Health Surveys (DHS) data from ten Central and Southeast Asian countries. Multilevel multinomial logistic regression models were used to compute odds ratios (ORs). PAFs adjusted for communality were calculated using adjusted ORs and prevalence estimates for each risk factor. This study included a weighted sample of 743,494 reproductive-age women. All the countries showed an increasing trend for obesity and a decreasing trend for underweight, except for the Maldives. The highest PAFs of obesity were associated with women who were married (PAF = 22.2%; 95% CI 22.1, 22.4), aged 35–49 years (PAF = 16.4%; 95% CI 15.5, 17.1), resided in wealthy households (PAF = 14.5%; 95% CI 14.4, 14.5), watched television regularly (PAF = 12.5%; 95% CI 12.1, 12.8), and lived in urban areas (PAF = 7.8%; 95% CI 7.7, 8.0). The combined PAF showed that these five risk factors were associated with 73.3% (95% CI 71.8, 74.9) of obesity among reproductive-age women. Interaction analysis between women’s education and household wealth revealed that having a secondary or higher level of education and residing in a wealthier household was associated with a lower risk of obesity (OR = 0.71, 95% CI 0.66, 0.76). The findings of this study suggest that, in order to address the rising rate of obesity among women in Asian countries, education and lifestyle modifications in urban areas should be a priority. Pakistan and the Maldives need to be a priority given the rapidly increasing trends in obesity and underweight subpopulations in their respective countries.

**Supplementary Information:**

The online version contains supplementary material available at 10.1038/s41598-024-73522-5.

## Background

Globally, approximately one-third of adults are overweight or obese, while around one in ten are underweight, with rates varying substantially across regions and countries^1^. The World Obesity Federation (WOF) predicts that by 2030, roughly one billion people worldwide will be living with obesity, including one in five women and one in seven men^2^. Estimates indicate a significant increase in the proportion of overweight and obese women in most low- and middle-income countries (LMICs). This increase is attributed to ongoing demographic and societal changes, including rising rural-to-urban migration, which lead to greater exposure to sedentary lifestyles, processed foods, and reduced access to traditional diets and physical activity^1,2^. The rising burden of obesity in women represents an enormous challenge with respect to non-communicable diseases (NCDs), increased healthcare costs, potential detrimental impacts on maternal and child health, reduced workforce productivity, and social and psychological implications^3,4^.

There is a tendency for an increasing prevalence of obesity among women in low-income populations with rapidly developing economies (e.g., China^5^ and Thailand^6^), and among the wealthier segments of society in slower developing economies (e.g., Bangladesh, Tanzania)^7^. Most LMICs in Southeast and Central Asia are currently in the mid-stages of economic development with a large segment of the population shifting towards less nutritious but more affordable foods and sedentary lifestyles^8,9^. Consequently, alongside pre-existing issues of malnutrition, there is a growing prevalence of overweight and obesity in specific sub-populations of women across the region^8–10^. This trend is an enormous challenge for local economies and fragile healthcare systems^9,11^. On regional and national levels, the dual challenges of malnutrition and obesity have created a dilemma, forcing policymakers to decide which issue to address first given the constraints of limited resources.

The epidemiological and nutrition transition may not solely attributed to economic development, but may also involve other demographic and societal changes, including rural-to-urban migration^12–14^. Asian countries appear to be following this trend, with a projected urbanization rate of 1.3% by 2030^13,14^. Around 40% of the urban population in Asia resides in mega cities, each with a population of over one million (e.g., Dhaka, Mumbai, Kolkata, Karachi, and Delhi). The population in Asian megacities is growing with a rate of > 2% per year, which also contributes to approximately 35% of the total population living in chronic poverty in urban slums^14^.

While increasing urbanization and commercialization may expose individuals and families to unhealthy diet and lifestyles, better education and higher income at the individual level should have an interrelated, counteracting effect on obesity^15^. Nationally representative studies, especially from middle-income Asian countries including China and Thailand, have in fact noted the protective effect of education and household income on female obesity^5,6^. Highly educated women and those who belonged to richer households are likely to have greater access to health information, be more health conscious, and have more choices regarding diet and exercise than lower-educated women from poorer households^16^. The existing evidence on trends in obesity and the relationship between education, household income and obesity among reproductive-age women in Central and Southeast Asia are limited and not well understood.

Policymakers in public health across Asia should examine the population-attributable fraction (PAF) of key risk factors of obesity among reproductive-age women^17^. This evidence is crucial for developing policy to curb intergenerational transmission of obesity^18^. Moreover, investigating the interaction between the socio-economic determinants of health and women’s nutritional status is further pertinent for the development of tailored prevention strategies. This will ultimately contribute to achieving the United Nations’ Sustainable Development Goal (SDG) target 3.4, which aims to reduce premature mortality from NCDs by one third through prevention and early intervention by 2030. Therefore, this study investigated the trends and PAF of obesity, and the interaction effects of education and wealth on obesity among reproductive-age women in ten Asian countries.

## Methods

### Study design and data sources

This cross-sectional study analysed the most recent (2012–2022) population-based data sets from the Demographic and Health Surveys (DHS) conducted in eight Southeast Asian countries and two Central Asian countries (Table 1). These countries encompassed Bangladesh, Cambodia, India, Kyrgyzstan, Maldives, Myanmar, Nepal, Pakistan, Tajikistan, and Timor-Leste. The exclusion of other countries in the region is due to surveys being restricted, unavailable, or lacking information on women’s body mass index (BMI). For each country, we retrieved data from available DHS conducted between 2000 and 2022 to analyse the trends of obesity, overweight, and underweight over the 22-year period.

**Table 1:** Nutritional status of reproductive-age women (15–49 years) from ten countries in Asia.

Countries	Survey year	Total population	Healthy weight* *n* (%)	Obesity* *n* (%)	Overweight* *n* (%)	Underweight* *n* (%)
Bangladesh	2017	18,328	10,211 (55.71)	1200 (6.55)	4738 (25.85)	2179 (11.89)
Cambodia	2021	9291	5660 (60.92)	522 (5.62)	2104 (22.65)	1005 (10.82)
India	2019	658,898	377,300 (57.26)	42,192 (6.40)	116,232 (17.64)	123,174 (18.69)
Kyrgyzstan	2012	6217	3480 (55.98)	798 (12.84)	1485 (23.89)	454 (7.30)
Maldives	2016	6667	2668 (40.02)	1290 (19.34)	1997 (29.94)	713 (10.69)
Myanmar	2015	12,189	7304 (59.92)	676 (5.54)	2329 (19.11)	1880 (15.42)
Nepal	2022	6980	4006 (57.40)	490 (7.02)	1548 (22.18)	935 (13.40)
Pakistan	2019	3722	1462 (39.27)	812 (21.81)	1132 (30.40)	317 (8.51)
Tajikistan	2017	9677	5379 (55.58)	1296 (13.39)	2291 (23.67)	712 (7.35)
Timor-Leste	2016	11,523	7331 (63.62)	172 (1.50)	958 (8.31)	3062 (26.57)
Total	2012–2022	743,494	424,802 (57.14)	49,449 (6.65)	134,814 (18.13)	134,430 (18.08)

*BMI cut-offs of < 18.5, 18.6–24.9, 25–29.9, and ≥ 30 kg/m2 are defined as underweight, healthy weight, overweight, and obesity, respectively.

The DHS program is funded by the United States Agency for International Development (USAID) and is implemented by Inner City Fund (ICF) International^19^. The Monitoring and Evaluation to Assess and Use Results (MEASURE) from the DHS studies were carried out by the respective Ministries of Health or governmental agencies, with support from ICF International, at regular intervals. The survey design and data are consistent, as they are based on standardized data collection methods and survey tools. The DHS gathers data on the demographics and health of individuals, encompassing topics such as maternal and child health, mortality, nutrition, and the social determinants of health^19^.

## Ethics

Ethical clearance was obtained for all DHS from the respective countries, whereas for this study, the requirement for informed consent was waived as publicly available data were used. This study followed the Strengthening the Reporting of Observational Studies in Epidemiology (STROBE) reporting guideline for cross-sectional studies.

## Sampling procedures and sample size

The DHS uses a two-stage stratified cluster sampling technique to select the study participants. In the first stage, the first administrative units (e.g., States and Regions) were stratified into urban or rural strata, followed by the selection of Enumeration Areas (EAs) in proportion to the population size of each cluster. In each selected EA, a complete census of households was conducted. In stage two, a fixed number of households were selected using the list of households as a sampling frame. Further details about the sampling design and questionnaire can be found in the country-specific MEASURE DHS reports.

For this study, the DHS survey data from the selected countries were pooled. The data were collected from eligible women, which included all females between the ages of 15 and 49 years who either lived in the households permanently or were present on the night before the survey. Women who were pregnant, or who had given birth in the two months preceding the survey were excluded to minimize measurement bias due to initial weight gain from pregnancy and childbirth.

## Outcome variables

We used the World Health Organization (WHO) BMI cut-offs of < 18.5, 25–29.9 and ≥ 30 kg/m2 to categorize underweight, overweight and obesity, respectively^20^. DHS used the SECA-874 digital scale to measure weight and the SECA-213 stadiometer to measure height. Trained survey staff obtained the measurements for a single time. BMI was rounded to the nearest hundredth decimal place.

## Explanatory variables

We considered several demographic and socio-economic variables including household wealth index, marital status, educational level, women’s age, number of children, current contraceptive use, place of residence, reading magazines, and watching television. The selection of the variables was informed by previous studies and the availability of relevant data. 8–10 Smoking, alcohol consumption, and women’s employment status were excluded from the analysis, either because they were not measured for each country or because they had a higher number of missing values. Table 2 provides the definitions for these explanatory variables.

The household wealth index represents a combined measurement of a household’s overall living standards. It is a composite measure of relative economic status estimated using household-level information on asset ownership and access to services from individual questionnaires^21^. In DHS, the household wealth index was computed using principal component analysis (PCA), considering various aspects like the possession of household amenities such as toilets, electricity, television, radio, fridge, and bicycle, as well as the availability of a source of drinking water and the type of flooring material used in the main house^22^. Each asset’s importance is calculated using factor scores which are standardized to have a mean of zero and a standard deviation of one. Based on whether a household owns a particular asset, standardized scores are assigned and summed up. Individuals are then ranked according to their household’s total score.

Since the relative importance of different assets varies across countries, PCA is a data-driven approach that allows the wealth index to be tailored to each country’s specific socioeconomic context. However, DHS uses wealth quintiles rather than the raw PCA scores, which allow for a comparative analysis of how wealth is distributed, how it correlates with health or education outcomes, and how inequality varies across countries^21^.

**Table 2:** Definitions of explanatory variables for obesity among women of reproductive age in ten Asian countries.

Variables	Definitions
Household wealth index	Grouped as ‘1’ = ‘poor or middle’, ‘2’ = ‘rich’
Education	Grouped as ‘1’ = ‘no schooling or low education’, or ‘2’ = ‘secondary education or higher’
Place of residence	Grouped as ‘1’ = ‘rural’, or ‘2’ = ‘urban’
Marital status	Grouped as ‘1’ = ‘Unmarried or formerly married’, or ‘2’ = ‘Currently married’
Current contraceptive use	Grouped as ‘1’ = ‘no’, or ‘2’ = ‘yes’
Women’s age	Grouped as ‘1’ = ‘15–34 years’, or ‘2’ = ‘35–49 years’
Number of children	Grouped as ‘1’ = ‘<4’, or ‘2’ = ‘>4’
Reading magazines	Grouped as ‘1’ = ‘no’, or ‘2’ = ‘yes’
Watching TV	Grouped as ‘1’ = ‘no’, or ‘2’ = ‘yes’

### Statistical analysis

The initial analyses involved calculating frequencies and percentages to provide an overview of the study population from the pooled data. To analyse the trends of obesity, overweight, and underweight, we calculated the prevalence, along with the corresponding 95% confidence intervals (CI), for each country from 2000 to 2022.

Multilevel multinomial logistic regression models were used in the pooled data to compute odds ratios (ORs) with 95% confidence intervals (CIs) for the risk factors for obesity, overweight and underweight (Model 1). Healthy weight groups were indicated as a reference category. The multi-level analysis was selected to account for the hierarchical structure of the data, where reproductive-age women [level I] were nested within clusters [level II]), consistent with previously published studies^23,24^. This approach enables accurate calculation of standard errors for regression coefficients and addresses the dependence of observations within the same clusters (i.e., women in the same cluster are likely to have more similar nutritional statuses than those in different clusters)^25^.

Upon identifying the key risk factors associated with obesity, overweight, and underweight, our analysis involved the computation of PAFs using Miettinen’s formula. The choice of Miettinen’s formula was based on its ability to produce reliable estimates, particularly in the presence of confounding, when adjusted ORs are used^26^. The PAF serves as a metric indicating the proportion of obesity among reproductive age women in Asian countries that could potentially be mitigated by addressing the identified potentially modifiable risk factors within the population^27^. PAF was calculated using the following formula:

$$\:{P}{A}{F}\:=\:{P}{c}({O}{R}-1)/\:{O}{R}$$

where Pc is the prevalence of the modifiable risk factor among cases, and OR is the adjusted ORs of obesity, overweight, and underweight associated with the potentially modifiable risk factors^26,27^. Given the modifiable risk factors occur simultaneously within individuals, aggregating the PAF for each specific risk factor may lead to an overestimation of their combined effects^28,29^. Based on previously published studies, we employed communality weights to correct for the overlap of risk factors among participants^30^.

To calculate communalities, we initially computed the pairwise tetrachoric correlation among all potentially modifiable risk factors. Pairwise tetrachoric correlation is specifically designed for assessing the relationships between pairs of dichotomous variables (i.e., binary variables with two possible outcomes: ‘0/1’ or ‘yes/no’). In datasets with multiple binary variables, tetrachoric correlation is applied to each pair of variables separately. This pairwise approach is used to isolate the direct relationship between two variables without the influence of others^31^. Subsequently, a principal components analysis was conducted on the tetrachoric correlation matrix to extract a set of common factors that explain the relationships. The communality for each risk factor was determined by the sum of squares of the loadings in all principal components with an eigenvector greater than 1. The weighting of each risk factor was then carried out using the formula: We = 1 − communality. Following this, a combined PAF across the potentially modifiable risk factors was calculated using the specified formula:

$$\:{P}{A}{F}\:\left({c}{o}{m}{b}{i}{n}{e}{d}\right)=\:1-\prod\:_{{r}=1}^{{R}}(1\:-{W}{e}{P}{A}{F}{e})$$

Where ‘*e’* represents each modifiable risk factor, and ‘We’ represents the communality weight of each risk factor. Finally, we estimated the adjusted PAF for each risk factor using the formula:

$$adjusted~PAFe\, = \,(\left[ {PAFe\,/\,\sum \,PAFe} \right]\,*\,combined~PAF$$.

To test the interaction effect of women’s education and household wealth index on obesity, an interaction term of women’s education and household wealth index was included in the model, with education as a categorical variable (Supplementary Table 1). Multicollinearity was checked using ‘vif’ command and no significant results were evident. All statistical analyses were conducted using Stata version 18.0 (StataCorp, College Station, TX, USA) with ’svy’ command to adjust for effect of sampling and stratification, and the ’gsem’ function was used for multilevel multinomial models to account the effect of two stage sampling design in DHS survey data.

## Results

### Study participants

This study involved 743,494 reproductive-age women aged 15–49 years, with a mean age of 30.7 (± 9.9) years. Of the total women, 40.8% (303,280) resided in wealthy households, and 64.6% (480,588) attained secondary or higher level of education. A total of 71.2% (528,949) women were married, and 38.3% (284,493) were in the age group 35–49 years. A total of 63.4% (471,039) women had 1–4 children, and 51.1% (379,516) were using contraception methods. A total of 67.6% (502,311) women were from rural areas. About 33.5% (248,832) of women read magazines, while 73.5% (546,263) watched television (Table 3).

**Table 3:** Selected social determinants of health and nutritional status of reproductive-age women (15–49 years) from ten countries in Asia.

	Overall	Healthy weight	Obesity	Overweight	Underweight
	*N* = 743,494 n (%)	*N* = 424,801 n (%)	*N* = 49,449 n (%)	*N* = 134,814 n (%)	*N* = 134,431 n (%)
Household wealth index					
Poor	286,269 (38.5)	175,126 (41.2)	8175 (16.5)	33,447 (24.8)	69,521 (51.7)
Middle	153,945 (20.7)	89,227 (21.0)	9078 (18.4)	28,391 (21.1)	27,250 (20.3)
Rich	303,280 (40.8)	160,448 (37.8)	32,196 (65.1)	72,976 (54.1)	37,660 (28.0)
Women’s education					
No schooling	163,362 (22.0)	99,227 (23.4)	8312 (16.8)	27,460 (20.4)	28,362 (21.1)
Primary school	99,541 (13.4)	57,230 (13.5)	7031 (14.2)	20,186 (14.9)	15,093 (11.2)
Secondary and above	480,588 (64.6)	268,343 (63.1)	34,105 (69.0)	87,166 (64.6)	90,975 (67.7)
Marital status					
Not married	180,169 (24.2)	104,792 (24.7)	3437 (7.0)	11,572 (8.6)	60,368 (44.9)
Currently married	528,949 (71.2)	300,521 (70.7)	42,825 (86.6)	115,900 (86.0)	69,703 (51.8)
Formerly married	34,376 (4.6)	19,489 (4.6)	3187 (6.4)	7342 (5.4)	4359 (3.2)
Women’s age					
15–24 years	238,199 (32.0)	140,893 (33.2)	4674 (9.5)	16,628 (12.3)	76,003 (56.6)
25–34 years	220,803 (29.7)	131,280 (31.9)	14,120 (28.5)	44,032 (32.7)	31,370 (23.3)
35–49 years	284,493 (38.3)	152,629 (35.9)	30,654 (62.0)	74,153 (55.0)	27,057 (20.1)
Parity					
None	223,203 (30.0)	130,596 (30.8)	5896 (11.9)	18,409 (13.6)	68,302 (50.8)
1–4 children	471,039 (63.4)	265,606 (62.5)	39,958 (80.8)	106,602 (79.1)	58,874 (43.8)
>4 children	49,248 (6.6)	28,599 (6.7)	3592 (7.3)	9803 (7.3)	7255 (5.4)
Contraceptive use					
No	363,962 (48.9)	207,774 (48.9)	18,831 (38.1)	49,578 (36.8)	87,779 (65.3)
Yes	379,516 (51.1)	217,018 (51.1)	30,618 (61.9)	85,235 (63.2)	46,646 (34.7)
Place of residence					
Urban	241,183 (32.4)	128,791 (30.3)	25,139 (50.8)	55,725 (41.3)	31,529 (23.5)
Rural	502,311 (67.6)	296,011 (69.7)	24,310 (49.2)	79,089 (58.7)	102,901 (76.5)
Reading magazine					
No	494,653 (66.5)	288,858 (68.0)	28,675 (58.0)	84,797 (62.9)	92,324 (68.7)
Yes	248,832 (33.5)	135,941 (32.0)	20,774 (42.0)	50,012 (37.1)	42,106 (31.3)
Watching television					
No	197,218 (26.5)	119,632 (28.2)	7300 (14.8)	26,227 (19.5)	44,059 (32.8)
Yes	546,263 (73.5)	305,162 (71.8)	42,148 (85.2)	108,583 (80.5)	90,371 (67.2)

Data are n (%) and are weighted using denormalised individual Demographic Health Survey weights.

### Trends in obesity

**Fig. 1:**
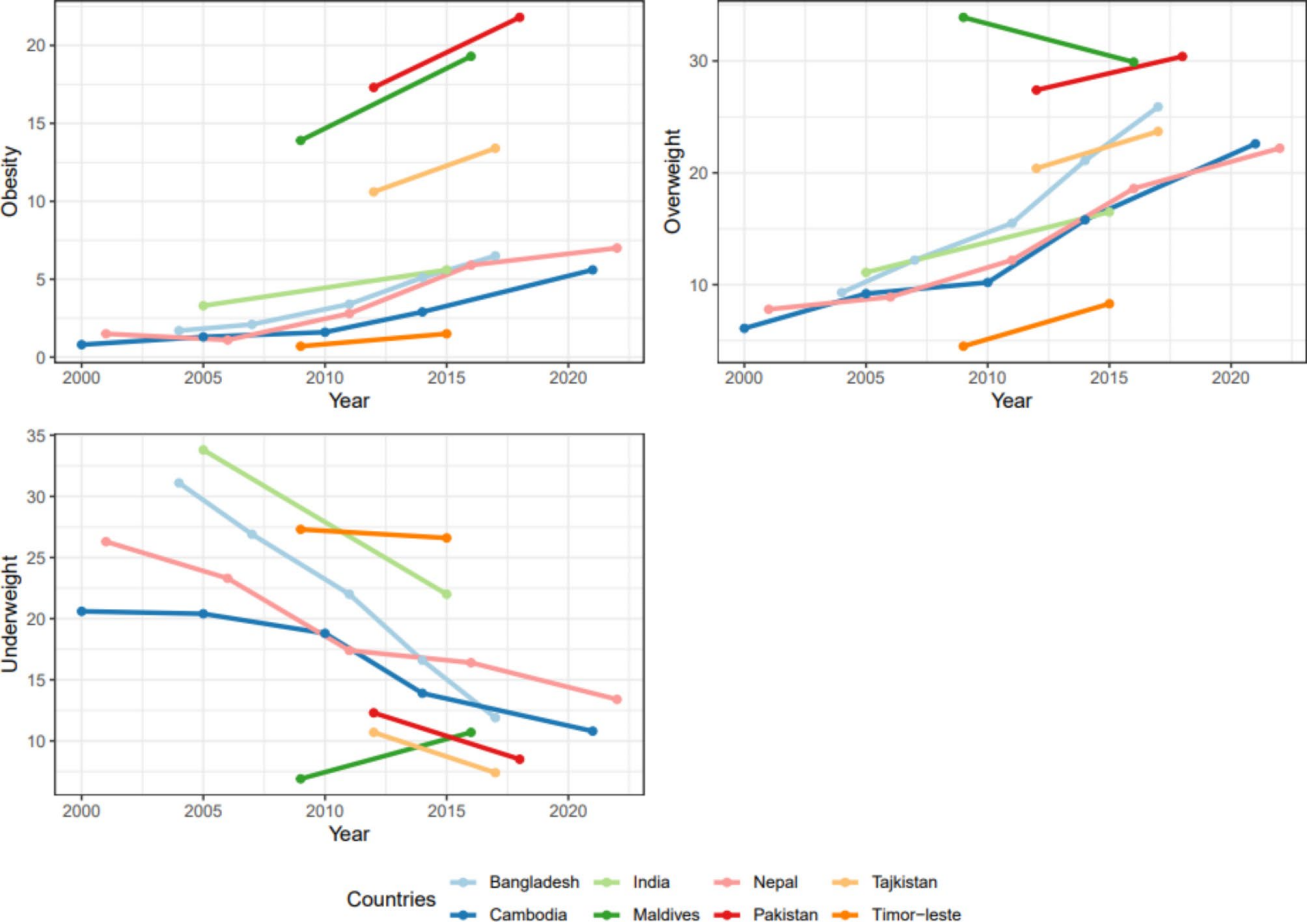
Trends (2000–2022) in obesity, overweight and underweight status among reproductive-age women aged 15–49 years of age from ten countries in Asia.

All ten South and Southeast Asian countries showed an upward trend in the obesity prevalence over the past two decades (Fig. 1). For example, in Pakistan, obesity increased from 17.3% in 2012 (95% CI: 15.2, 19.6) to 21.8% (95% CI: 19.6, 24.2) in 2018. A similar trend was observed in Maldives with obesity increasing from 13.9% (95% CI: 12.5, 15.4) in 2009 to 19.3% (95% CI: 18.1, 20.7) in 2016. All countries had a gradual increase in overweight and a gradual decrease in underweight prevalence, except for the Maldives. In Maldives, between 2009 and 2016, the prevalence of overweight decreased from 33.9% (95% CI: 32.1, 35.8) to 29.9% (95% CI: 28.4, 31.6), while the prevalence of underweight increased from 6.9% (95% CI: 5.9, 7.9) to 10.7% (95% CI: 9.7, 11.8).

### Population attributable fractions for obesity

The highest PAFs of obesity were associated with married women (PAF = 22.2%; 95% CI: 22.1, 22.4), those aged 35–49 years (PAF = 16.4%; 95% CI: 15.5, 17.1), individuals who resided in rich households (PAF = 14.5%; 95% CI: 14.4, 14.5), women who watched television on a regular basis (PAF = 12.5%; 95% CI: 12.1, 12.8), and for those who were from urban areas (PAF = 7.8%; 95% CI: 7.7, 8.0) (Table 4). The combined PAF showed that these five risk factors were associated with 73.3% (95% CI: 71.8, 74.9) of obesity among reproductive-age women. Additionally, these same five risk factors were associated with 58.5% (95% CI: 58.1, 60.5) of them being overweight (Supplementary Table 2).

**Table 4:** Population-attributable fractions for obesity among reproductive-age women aged 15–49 years from ten countries in Asia from 2012–2022.

Variables	Prevalence of risk factors in cases	OR (95% CI)	Unadjusted PAF% (95% CI)	Adjusted* PAF% (95% CI)
Household wealth index				
Rich	65.1 (64.3, 65.9)	2.16 (2.11, 2.22)	34.97 (33.76, 36.18)	14.44 (14.40, 14.46)
Poor or medium	34.9 (34.1, 33.7)	Ref	Ref	Ref
Education				
Secondary or higher	69.0 (68.3, 69.7)	1.12 (1.09, 1.15)	7.47 (5.72, 9.21)	3.08 (2.44, 3.68)
No or low education	31.0 (30.4, 31.7)	Ref	Ref	Ref
Place of residence				
Urban	50.8 (49.7, 52.0)	1.59 (1.54, 1.63)	18.83 (17.51, 20.17)	7.78 (7.68–8.06)
Rural	49.2 (48.0, 50.3)	Ref	Ref	Ref
Concentrative device				
Yes	61.9 (61.2, 62.7)	1.00 (0.97, 1.02)	–	–
No	38.1 (37.3, 38.8)	Ref	Ref	Ref
Marital status				
Currently married	86.6 (86.1, 87.1)	2.66 (2.57, 2.75)	53.99 (52.57, 55.38)	22.29 (22.13, 22.42)
Unmarried or separated	13.4 (12.9, 13.9)	Ref	Ref	Ref
Women’s age				
35–49 years	62.0 (61.4, 62.6)	2.80 (2.73, 2.86)	39.84 (38.91, 40.76)	16.45 (15.46, 17.07)
15–34 years	38.0 (37.4, 38.7)	Ref	Ref	Ref
Parity				
>4 children	7.3 (6.9, 7.7)	1.00 (0.96, 1.04)	–	–
<4 children	92.7 (92.4, 93.1)	Ref	Ref	
Reading magazine				
Yes	42.0 (41.2, 42.8)	1.22 (1.19, 1.25)	7.53 (6.52, 8.55)	3.11 (2.78, 3.42)
No	58.0 (57.2, 58.8)	Ref	Ref	Ref
Watching television				
Yes	85.2 (84.7, 85.8)	1.55 (1.50, 1.60)	30.23 (28.36, 32.06)	12.48 (12.09, 12.81)
No	14.8 (14.2, 15.3)	Ref	Ref	Ref

PAF: population attributable fraction; OR: odds ratio; CI: Confidence Interval.

* Weighted PAF is the relative contribution of each risk factor to the overall PAF when adjusted for communality.

### Interaction effect of women’s education and wealth on obesity

The interaction analysis between women’s education and household wealth index revealed that, in rich households, women who completed secondary or higher level of education had a lower risk of obesity (OR = 0.71, 95% CI: 0.66, 0.76) compared to those with no formal education (Supplementary Table 2, Model 2). In wealthy households, women who completed secondary or higher level of education exhibited a higher risk of underweight (OR = 1.52, 95% CI: 1.44, 1.61) compared to those with no formal education.

## Discussion

This is the first study to investigate the trends and PAF of obesity, as well as the effects of education-wealth interaction on obesity among reproductive-age women in ten Asian countries. The findings from our study identified a rising trend in obesity. All the countries showed an increasing trend for overweight, and a decreasing trend for underweight, except for the Maldives. A total of 73.3% of obesity among reproductive age women was associated with five risk factors: being married, age ≥ 35 years, residing in wealthy households, regular television watching and living in urban areas.

The computation of PAFs for obesity provides an opportunity to guide resource distribution, particularly in countries, such as Pakistan and the Maldives, that are dealing with a substantial burden of obesity. The use of nationally representative DHS datasets further strengthens the applicability of our findings to the broader regional context. The risk factors investigated in this study hold broader implications for preventing obesity in women and improving women’s health and wellbeing. Programs promoting nutritional education and healthier lifestyles, prioritizing women in urban areas, particularly those residing in affluent households who engage in unhealthy eating habits and physical inactivity, are cost effective investments for Central and Southeast Asian countries^32,33^. It is estimated that for each 1 USD invested in preventing obesity, up to 5.6 USD will be returned in economic benefits^33^.

Obesity remains a significant challenge among wealthier segments of society, particularly within urban populations in Central and Southeast Asian countries^7^. The risk also tends to increase among women who are married, older, or frequently engage with media such as magazines or television. Addressing the obesity epidemic among Asian women of childbearing age requires a multifaceted approach. Interventions such as promoting healthier diets and reducing sedentary behaviours through public health campaigns, increasing access to affordable nutritious foods, and policies that regulate food advertising and promote physical activity in urban areas may help combat the growing obesity epidemic^34^. In some Asian cultures, women are often expected to prioritize caregiving and household responsibilities, which can limit their ability to access and engage in physical activity. Familial and peer support strategies are needed to help women overcome traditional gender roles and create an enabling environment that sustains their health-behaviour changes^35^.

The effect of interaction between education and household wealth on women’s obesity risk merits closer attention, as this has not been extensively discussed previously^36,37^. The education-obesity relationship is not uniform in all Asian societies due to the varying degree of progress in educational development, economic development, and globalization between the countries^38,39^. In addition to experiencing rapid economic growth, middle- to high-income Asian countries have introduced policies and initiatives aimed at improving women’s access to higher education, positioning education as a modifiable factor for obesity prevention within the broader socio-economic landscape^40^. However, policies and interventions for obesity prevention across LMICs in Asia have not prioritised long term measures, such as promoting women to complete secondary education^41^.

The positive associations between living in urban areas, household wealth, sedentary behaviours and obesity can still be counteracted with encouraging women to complete secondary level or higher education, as shown by the experience of middle to high income Asian countries^5,6,39,42,43^. Women with higher education have the ability to challenge pre-existing gender norms, and make informed health and behavioural choices with respect to nutritional choices and lifestyle changes^44^. The importance of women completing a higher education should be acknowledged not only for preventing obesity among women and improving gender equality and the health of the entire family but also for the future economic development of an entire nation.

Asian megacities, comprising 35% of the population living in slums, demand enhanced public services such as access to healthier foods and clean water, yet improvements in these crucial areas have unfortunately not kept pace with the rapid urbanization^14^. Consequently, a considerable segment of general women’s population as well as women from urban areas, for instance in Timor-Leste, India, Pakistan, and the Maldives, is malnourished and faces an increased risk of infectious diseases due to poorer water, hygiene, and sanitation conditions. To tackle the double burden of nutritional problems in women, especially in impoverished areas, poverty reduction measures (e.g., employment training and micro-financing), as well as encouragement of local food production and consumption can be instrumental (Supplementary Table 3)^45^.

Veblen’s 1899 hypothesis^46^ associating female desire for thinness with higher social classes seems to be not influenced by wealth alone but rather due to the interaction between higher education and household wealth in Asian countries. It is true that the beauty industry and social media, to which affluent and educated women have greater access, have perpetuated a social preference for thinness over plumpness as a feminine ideal. However, it is important to recognize that while addressing malnutrition in women on a broader scale, body image concerns and eating disorders are typically more prevalent in rich women is a false stereotype and that nutritional disorders can be present across a wide range of socioeconomic backgrounds^47^.

### Strengths and limitations of the study

This study has strengths. The large sample of 743,494 reproductive-age (15–49 years) women from ten Asian countries allowed us to examine PAFs for key risk factors and analyse interaction between education and wealth on obesity.

This study also had limitations. First, the use of cross-sectional data presents difficulties in establishing a temporal relationship between covariates and the outcome variable. Second, BMI as a measure of obesity does not reflect the location or amount of body fat of women. However, studies suggest that BMI is correlated to more direct measures of body fat, such as underwater weighing and dual-energy x-ray absorptiometry^48^. Third, we used WHO BMI cut-offs (< 18.5, 25–29.9 and ≥ 30 kg/m^2)^ to categorize underweight, overweight and obesity. However, this cut-off may not be equally applicable across different populations due to differences in body composition and stature^49^. Forth, we merged widowed or divorced women as formerly married and did not test their independent association with obesity as there might be strong socio-cultural implications of these factors for women in traditional societies^50^.

Fifth, the study was limited by a lack of data on key behavioural factors, including dietary habit and physical activity, as the DHS did not collect information on these variables. Sixth, most of the explanatory variables were measured based on self-reported questionnaires and which could be a source of recall bias. Last, PAF estimates rely on particular assumptions, involving causality, the independence of modifiable risk factors, and consistent associations over time^51^. However, these assumptions might prove unrealistic due to the intricate interplay of socio-economic, cultural, healthcare, and behavioural factors associated with women’s nutritional status. Despite this complexity, PAFs offer a straightforward and intuitive metric that can supplement other methodologies in pinpointing modifiable risk factors suitable for policy intervention.

## Conclusion

Central and Southeast Asian countries are experiencing a rising trend in female obesity, accompanied by a gradual reduction in underweight. A total of 73.3% of obesity among reproductive age women was associated with being married, age ≥ 35 years, residing in rich households, regular television watching and living in urban areas. Completing secondary or higher level of education exhibits a lower risk of obesity, particularly in wealthy households. Considering the increasing rate of urbanization in Asia, future interventions to prevent obesity should prioritize women in urban areas, particularly those residing in affluent households, with lower educational attainment, and engaging in unhealthy eating habits and physical inactivity. Preventing obesity should be a regional priority if SDG target 3.4 is to be achieved by 2030. Pakistan and the Maldives need to be a priority given the rapidly increasing trends in obesity and underweight subpopulations in their respective countries.

## Electronic supplementary material

Below is the link to the electronic supplementary material.

Supplementary Material 1

## Data Availability

The datasets supporting the conclusions of this article are available in the DHS repository, [https://dhsprogram.com/data/available-datasets.cfm]. The DHS provides open access to survey data files for legitimate academic research purposes. To initiate the download process, registration is mandatory. Researchers are required to provide their contact information, research title, and a brief description of the proposed analysis. Approval for dataset access is typically confirmed via email. It is important to note that these datasets are third-party resources and not under the ownership or collection of the authors, who possess no special access privileges.

## Abbreviations

BMI: Body Mass Index
CI: Confidence interval
DHS: Demographic health survey
ICF: Inner city fund
LMICs: Low- and middle-income countries
MEASURE: Monitoring and evaluation to assess and use results
NCDs: Non-communicable diseases
OR: Odds ratio
PAF: Population attributable fraction
SDG: Sustainable development goals
USAID: United States Agency for International Development
WOF: World obesity federation
