# Supplementary material for: Trends and social determinants of the obesity epidemic among reproductive-age women in ten Asian countries
Source: Sci Rep. 2024 Sep 29;14:22545. doi: 10.1038/s41598-024-73522-5 (PMC11439913; doi:10.1038/s41598-024-73522-5)
Supplement: Supplementary file 1 — Supplementary Material 1 [file 41598_2024_73522_MOESM1_ESM.docx]

**Supplementary files**

Supplementary Table 1. Population-attributable fractions for being overweight among reproductive-age women aged 15-49 years from ten countries in Asia from 2012–2022.

| **Variables** | **Prevalence of risk factors in cases** | **OR (95% CI)** | **Unadjusted**  **PAF% (95% CI)** | **Adjusted***  **PAF% (95% CI)** |
| --- | --- | --- | --- | --- |
| Household wealth index |  |  |  |  |
| Rich | 54.1 (53.5, 54.8) | 1.58 (1.56, 1.61) | 19.93 (19.14, 20.72) | 9.37 (9.39, 9.76) |
| Poor or medium | 45.9 (45.2, 46.5) | Ref | Ref | Ref |
| Education |  |  |  |  |
| Secondary or higher | 64.7 (64.2, 65.1) | 1.09 (1.07, 1.11) | 5.31 (4.27, 6.35) | 2.50 (2.09, 2.99) |
| No or low education | 35.3.0 (34.9, 35.8) | Ref | Ref | Ref |
| Place of residence |  |  |  |  |
| Urban | 41.3 (40.4, 42.3) | 1.28 (1.25, 1.30) | 8.93 (8.12, 9.75) | 4.19 (3.98-4.60) |
| Rural | 58.7 (57.7, 59.6) | Ref | Ref | Ref |
| Concentrative device |  |  |  |  |
| Yes | 63.2 (62.7, 63.7) | 1.11 (1.09, 1.13) | 6.29 (5.35, 7.23) | 2.96 (2.63-3.41) |
| No | 36.8 (36.3, 37.3) | Ref | Ref | Ref |
| Marital status |  |  |  |  |
| Currently Married | 86.0 (85.7, 86.3) | 2.27 (2.22, 2.31) | 48.06 (47.14, 48.97) | 22.59 (23.12, 23.08) |
| Unmarried or separated | 14.0 (12.9, 13.9) | Ref | Ref | Ref |
| Women’s age |  |  |  |  |
| 35–49 years | 55.0 (54.6, 55.4) | 2.07 (2.04, 2.10) | 28.46 (27.87, 29.06) | 13.38 (13.00, 13.69) |
| 15–34 years | 45.0 (44.6, 45.4) | Ref | Ref | Ref |
| Parity |  |  |  |  |
| >4 children | 7.3 (7.1, 7.5) | 0.91 (0.89, 0.93) | – | – |
| <4 children | 92.7 (92.5, 93.0) | Ref | Ref |  |
| Reading magazine |  |  |  |  |
| Yes | 37.1 (36.6, 37.7) | 1.13 (1.11, 1.15) | 4.29 (3.68, 4.90) | 2.02 (1.81, 2.31) |
| No | 62.9 (62.4, 63.5) | Ref | Ref | Ref |
| Watching television |  |  |  |  |
| Yes | 80.6 (80.1, 81.0) | 1.31 (1.28, 1.33) | 18.92 (17.75, 20.07) | 8.89 (8.70, 9.46) |
| No | 19.5 (19.1, 19.9) | Ref | Ref | Ref |

PAF: population attributable fraction; OR: odds ratio; CI: Confidence Interval

* Weighted PAF is the relative contribution of each risk factor to the overall PAF when adjusted for communality

Supplementary Table 2. Association between covariates and nutritional status among reproductive-age women aged 15-49 years from ten countries in Asia.

| **Variables** | **Obesity** | | **Overweight** | | **Underweight** | |
| --- | --- | --- | --- | --- | --- | --- |
|  | Model 1 | Model 2 | Model 1 | Model 2 | Model 1 | Model 2 |
|  | OR (95% CI) | OR (95% CI) | OR (95% CI) | OR (95% CI) | OR (95% CI) | OR (95% CI) |
| Household wealth index |  |  |  |  |  |  |
| Poor | 1.00 | 1.00 | 1.00 | 1.00 | 1.00 | 1.00 |
| Middle | 1.76 (1.71-1.82) | 2.03 (1.91-2.16) | 1.51 (1.48-1.54) | 1.59 (1.54-1.65) | 0.86 (0.84-0.87) | 0.74 (0.71-0.76) |
| Rich | 2.95 (2.86-3.05) | 3.56 (3.36-3.78) | 1.96 (1.92-2.00) | 2.15 (2.07-2.23) | 0.72 (0.71-0.74) | 0.50 (0.48-0.53) |
| Women’s education |  |  |  |  |  |  |
| No schooling |  | 1.00 |  | 1.00 |  | 1.00 |
| Primary school | 1.23 (1.18-1.27) | 1.26 (1.19-1.35) | 1.15 (1.13-1.18) | 1.19 (1.15-1.23) | 0.88 (0.86-0.90) | 0.85 (0.83-0.88) |
| Secondary and above | 1.30 (1.26-1.35) | 1.61 (1.53-1.70) | 1.23 (1.20-1.25) | 1.31 (1.28-1.35) | 0.80 (0.78-0.81) | 0.73 (0.71-0.74) |
| Household wealth X Education |  |  |  |  |  |  |
| Middle X Primary school | - | 0.93 (0.84-1.02) | - | 0.93 (0.88-0.98) | - | 1.08 (1.02-1.15) |
| Middle X secondary and above | - | 0.75 (0.69-0.81) | - | 0.90 (0.86-0.94) | - | 1.23 (1.17-1.28) |
| Rich X Primary school | - | 0.91 (0.84-0.99) | - | 0.92 (0.87-0.97) | - | 1.27 (1.18-1.37) |
| Rich X Secondary and above | - | 0.71 (0.66-0.76) | - | 0.86 (0.82-0.90) | - | 1.52 (1.44-1.61) |
| Marital status |  |  |  |  |  |  |
| Not married | 1.00 | 1.00 | 1.00 | 1.00 | 1.00 | 1.00 |
| Currently married | 2.51 (2.36-2.67) | 2.51 (2.36-2.67) | 1.97 (1.90-2.05) | 1.97 (1.90-2.05) | 0.65 (0.63-0.67) | 0.65 (0.63-0.67) |
| Formerly married | 2.25 (2.09-2.42) | 2.25 (2.09-2.42) | 1.72 (1.65-1.80) | 1.72 (1.65-1.80) | 0.71 (0.68-0.74) | 0.71 (0.68-0.74) |
| Women’s age |  |  |  |  |  |  |
| 15–24 years | 1.00 | 1.00 | 1.00 | 1.00 | 1.00 | 1.00 |
| 25–34 years | 2.35 (2.24-2.45) | 2.35 (2.24-2.45) | 2.11 (2.06-2.16) | 2.11 (2.06-2.16) | 0.53 (0.52-0.54) | 0.53 (0.52-0.54) |
| 35–49 years | 4.77 (4.56-4.99) | 4.77 (4.56-4.99) | 3.37 (3.29-3.46) | 3.37 (3.29-3.46) | 0.36 (0.35-0.37) | 0.36 (0.35-0.37) |
| Parity |  |  |  |  |  |  |
| None | 1.00 | 1.00 | 1.00 | 1.00 | 1.00 | 1.00 |
| 1–4 children | 1.15 (1.09-1.21) | 1.15 (1.09-1.21) | 1.10 (1.06-1.13) | 1.10 (1.06-1.13) | 1.02 (0.99-1.05) | 1.02 (0.99-1.05) |
| >4 children | 1.19 (1.11-1.27) | 1.19 (1.11-1.27) | 1.02 (0.98-1.06) | 1.02 (0.98-1.06) | 1.24 (1.19-1.30) | 1.24 (1.19-1.30) |
| Contraceptive use |  |  |  |  |  |  |
| No | 1.00 | 1.00 | 1.00 | 1.00 | 1.00 | 1.00 |
| Yes | 0.87 (0.85-0.89) | 0.87 (0.85-0.89) | 0.98 (0.97-1.02) | 0.98 (0.97-1.02) | 0.89 (0.88-0.91) | 0.89 (0.88-0.91) |
| Place of residence |  |  |  |  |  |  |
| Urban | 1.00 | 1.00 | 1.00 | 1.00 | 1.00 | 1.00 |
| Rural | 0.67 (0.65-0.69) | 0.67 (0.65-0.69) | 0.83 (0.81-0.84) | 0.83 (0.81-0.84) | 1.13 (1.10-1.15) | 1.13 (1.10-1.15) |
| Reading magazine |  |  |  |  |  |  |
| No | 1.00 | 1.00 | 1.00 | 1.00 | 1.00 | 1.00 |
| Yes | 1.21 (1.18-1.24) | 1.21 (1.18-1.24) | 1.11 (1.09-1.13) | 1.11 (1.09-1.13) | 0.94 (0.92-0.95) | 0.94 (0.92-0.95) |
| Watching television |  |  |  |  |  |  |
| No | 1.00 | 1.00 | 1.00 | 1.00 | 1.00 | 1.00 |
| Yes | 1.40 (1.35-1.44) | 1.40 (1.35-1.44) | 1.21 (1.19-1.23) | 1.21 (1.19-1.23) | 0.92 (0.90-0.93) | 0.92 (0.90-0.93) |

*Model 2 is adjusted for all explanatory variables in Model 1 plus the interaction between household wealth index and women’s education.*

Supplementary Table 3. Population-attributable fractions for being underweight among reproductive-age women aged 15-49 years from ten countries in Asia from 2012–2022.

| **Variables** | **Prevalence of risk factors in cases** | **OR (95% CI)** | **Unadjusted**  **PAF% (95% CI)** | **Adjusted***  **PAF% (95% CI)** |
| --- | --- | --- | --- | --- |
| Household wealth index |  |  |  |  |
| Rich | 28.0 (27.4, 28.7) | Ref | Ref | Ref |
| Poor or medium | 72.0 (71.4, 72.6) | 1.32 (1.30, 1.35) | 17.61 (16.53, 18.69) | 7.48 (7.10, 8.21) |
| Education |  |  |  |  |
| Secondary or higher | 67.7 (67.2, 68.1) | Ref |  |  |
| No or low education | 32.3 (31.9, 32.8) | 1.00 (0.98, 1.01) | – | – |
| Place of residence |  |  |  |  |
| Urban | 23.5 (40.4, 42.3) | Ref | Ref | Ref |
| Rural | 76.7 (57.7, 59.6) | 1.14 (1.12, 1.16) | 9.51 (8.08, 10.09) | 4.04 (3.96, 4.39) |
| Concentrative device |  |  |  |  |
| Yes | 34.7 (34.3, 35.1) | Ref |  |  |
| No | 65.3 (64.9, 65.7) | 1.73 (1.71, 1.76) | 27.62 (26.91, 28.32) | 11.72 (12.00, 13.96) |
| Marital status |  |  |  |  |
| Currently Married | 51.9 (51.4, 52.3) | Ref | Ref | Ref |
| Unmarried or separated | 48.2 (47.7, 48.6) | 1.19 (1.15, 1.23) | 7.63 (6.14, 9.09) | 3.24 (3.01, 4.48) |
| Women’s age |  |  |  |  |
| 35–49 years | 55.0 (54.6, 55.4) | Ref |  |  |
| 15–34 years | 45.0 (44.6, 45.4) | 2.00 (1.96, 2.03) | 39.91 (39.07, 40.74) | 19.94 (19.15, 20.08) |
| Parity |  |  |  |  |
| >4 children | 5.4 (5.2, 5.6) | Ref |  |  |
| <4 children | 94.6 (94.4, 94.8) | 0.88 (0.85, 0.90) | – | – |
| Reading magazine |  |  |  |  |
| Yes | 31.3 (30.8, 31.8) | Ref | Ref | Ref |
| No | 68.7 (68.2, 69.2) | 1.02 (1.00, 1.04) | 1.34 (0.23, 2.45) | 0.57 (0.12, 1.21) |
| Watching television |  |  |  |  |
| Yes | 67.2 (66.7, 67.7) | Ref | Ref | Ref |
| No | 32.8 (32.3, 33.3) | 1.12 (1.10, 1.13) | 3.41 (2.91, 3.92) | 1.45 (1.43, 1.93) |

PAF: population attributable fraction; OR: odds ratio; CI: Confidence Interval

* Weighted PAF is the relative contribution of each risk factor to the overall PAF when adjusted for communality
